# Large Cyst of Kidney—Nephrectomy—Recovery*Read before the Clinical Society of Louisville, July, 1893.

**Published:** 1893-09

**Authors:** L. S. McMurtry

**Affiliations:** Louisville, Ky.


### LARGE CYST OF KIDNEY—NEPHRECTOMY—RECOVERY.*

BY L. S. MCMURTRY, M. D., LOUISVILLE, KY.

The extension of the field of abdominal and pelvic surgery and the splendid results attained in recent years have become so familiar to the profession that isolated cases have ceased to be of special interest. The following case, however, is somewhat unique and illustrates so many important practical points that I deem it worthy of special report and discussion.

On June 25th, I was asked by my friend, Dr. Henry E. Pelle, to see a patient with him in the west end of the city. The patient is thirty-two years of age and unmarried, and pre-

* Read before the Clinical Society of Louisville, July, 1893.

sented the following history and symptoms: About three years ago she noticed an enlargement of the abdomen toward the left side. It gave her no discomfort at first but she observed that it steadily increased in size. During the past year it had grown rapidly and had now assumed the proportions of a large abdominal tumor, giving her abdomen the general contour and appearance of a woman near the full term of utero-gestation except the tumor was larger toward the left side. Her general health was excellent except that she was losing flesh and suffering more and more with pressure symptoms. During the past few months she had perceptibly emaciated, and was unable to eat a full meal on account of the discomfort following. Her activity was interferred with also, being unable to discharge her duties easily on account of the weight and pressure of the tumor. Physical examination of the abdomen disclosed the typical signs of a monocystic ovarian tumor. It occupied the general abdominal cavity as well as the pelvis, had pushed the uterus backward into Douglas' space, thinned the abdominal walls and fluctuated distinctly upon percussion. All the symptoms were characteristic of a cyst: Abdominal dropsy was readily excluded. She had expressed her desire to Dr. Pelle for permanent relief and he had made known to her the necessities for operative interference. She was at once placed upon preparatory treatment and went to the infirmary to undergo the operation.

The operation was performed on July 3d, at 9 a. m. Besides the assistants and nurses, Dr. Pelle and Dr. R. C. Chenault, of this city, were present. A free incision was made in the median line between the umbilicus and pubes as in ovariotomy. On dividing the parietal peritoneum I was puzzled by finding another movable layer of peritoneum covering the cyst. My first thought was that it might be an ovarian cyst which had grown in the leaflets of the broad ligament so as to present such a duplicate fold of the serous membrane, but passing my two fingers down in front of the cyst into the pelvis I found both ovaries in their proper position and quite normal in every respect. This demonstrated that the tumor had its origin in the post-peritoneal space and had carried the peritoneum in front of it in its development. Following the outline of the tumor with my hand I soon traced its attachments to the left lumbar region and satisfied myself that I was dealing with a large cyst of the left kidney. Announcing this to the anæsthetist, Dr. J. W. Guest, he immediately substituted chloroform for ether which he had been using up to this stage of the operation, and continued to use chloroform throughout the remainder of the operation. Cystic degeneration of the kidney usually presents a large number of small cyst clustered together, and hydronephrosis, which is the result of an obstructed ureter, is limited for the most part to the pelvis of the kidney with varying degrees of renal structures preserved. This cyst, however, presented the pearly hue of an ovarian cyst, and as the specimen presented herewith shows, consists of the capsule converted into a large single cyst. The capsule was distended and rendered very thin; it was easily punctured with the trocar and the contents emptied into the tub beneath the table. I presume that it contained about one gallon of clear serous fluid. After emptying the cyst I found quite a task presented in separating the sac from its attachments to the peritoneum of the left lumbar region and in freeing it from the spleen, to which it was firmly attached and which appeared as a cap to the tumor. In separating the spleen, although observing the utmost care, I injured the capsule of that organ and denuded a considerable surface upon its inferior border. This started a capillary hemorrhage which persisted throughout the remainder of the operation, and for three days afterward. After freeing the cyst in all directions, I tied the ureter and divided it. I then secured the renal vessels with a strong silk ligature, ligating them in two parts, and cut away the cyst. I then closed with fine silk sutures the opening in the posterior layer of the peritoneum through which I had delivered and removed the diseased kidney. An examination of the kidney on the right side at this stage of the operation demonstrated that it was of normal consistency but very appreciably enlarged, which I attribute to the additional work thrown upon it by the progressive crippled condition of the other kidney. After cleaning the abdomen thoroughly and securing all bleeding points, I found the denuded surface of the spleen still oozing, and that the sponge packing I had applied had done little to arrest it. The bleeding was not great and not active, but consisted of a steady oozing of bright blood. In order to deal with the pedicle and secure thoroughly the renal vessels, I had made a transverse incision in the abdominal parietes at right angles to the median incision and extended this into the left hypochondrium. I now closed the abdomen by suturing carefully both incisions, being careful to adjust accurately the severed rectus muscle end to end, and having placed a glass drainage tube through the lower angle of the median incision with the point resting in Douglas' space, where all oozing fluid would naturally gravitate. The usual dressing was applied and the patient put to bed in good condition. There was no sweating or other symptom of shock, the pulse being 104 and of good volume.

The convalescence has been exceptionally easy and uneventful. The secretion of urine was quite free and indicative of excellent kidney function. This has continued without interruption throughout her convalescence, and as it is evident from the specimen that all secretory tissue had long been destroyed in the diseased kidney its fellow had evidently assumed compensatory structural and functional activity. The drainage was quite free for the first twenty-four hours, the bright color of the blood showing that it came from the spleen. This gradually ceased and the tube was removed on the fourth day. The pulse remained under 100, and the highest temperature recorded was 99.6 F. The large double incision united throughout by first intention, and the stitches were removed on the ninth day. As a precaution against hernia after such extensive incision of the abdominal walls, I have kept the patient upon the back, and will insist upon keeping her in bed for three weeks, by which time organization and consolidation of the incision will be complete. Her appetite is excellent and all her digestive and eliminative functions seem to be carried on perfectly, which I interpret as an indication of good compensatory power on the part of the right kidney and a guarantee of restoration to perfect health. This woman has naturally a strong constitution, has been accustomed to health-giving habits of frugality and industry, with temperate habits.

I herewith present for examination the sac of this large tumor, which, in its lower part, still retains an outline of the form of the kidney. The fluid contaiued in this cyst was not urine, but a perfectly clear fluid, such as we find in a typical par-ovarian cyst. It was an illustrative case of general cystic degeneration of the kidney, the capsule of the kidney forming a strong cyst-wall just as obtains in large ovarian cysts.

The practical lessons to be deduced from this experience are: First, that in the diagnosis of abdominal tumors a large experience and pains-taking investigation cannot determine infallibly the exact character of even typical illustrations of ovarian tumors. The greater frequency of ovarian cysts alone determines a presumptive diagnosis between ovarian cyst and renal cyst. The exploratory incision is at last the means of positive diagnosis.

Second. In operations presumably for simple uncomplicated ovarian tumors, the safety of the patient demands that the operator be qualified and prepared to deal with unexpected complications and to do any operation known in abdominal surgery.

Third. The results of nephrectomy as shown by recent work place this class of tumors within the limit of safe surgical procedures.

Fourth. In dealing with large renal growths, and where nephrectomy may of necessity follow nephrotomy, the lateral abdominal incision in the semi-lunar line is preferable to the lumbar incision.

---

TO BE BURIED ALIVE.—A "mind reader" in Illinois has published his intention to be buried alive and remain in the ground while a cup of barley is being grown over his grave. He has read of the Man with the Broken Ear, and thinks he can give him points on his peculiar specialty.—*Ex.*

